# 3-Acetyl-4-hydroxy­phenyl acrylate

**DOI:** 10.1107/S1600536809032176

**Published:** 2009-08-29

**Authors:** V. Azeezaa, G. Usha, Sundari Bhaskaran, A. Anthonysamy, S. Balasubramanian

**Affiliations:** a Department of Physics, Queen Mary’s College (Autonomous), Chennai 600 004, India; b Department of Inorganic Chemistry, University of Madras, Guindy Campus, Chennai 600 025, India

## Abstract

In the title compound, C_12_H_12_O_4_, the hydr­oxy O and the C and O atoms of the acetyl group are almost coplanar [maximum deviation = 0.0356 (1) Å] with the benzene ring. The dihedral angle between the benzene ring and the plane through the non-H atoms of the methacrylo­yloxy group is 86.1 (1)°. In the crystal structure, mol­ecules are linked by two C—H⋯O hydrogen bonds, forming dimers with graph-set descriptor *R*_2_^2^(16). A strong intra­molecular O—H⋯O hydrogen bond is also observed.

## Related literature

For reference bond-length data, see: Allen *et al.* (1987 ▶). For graph-set notation, see Bernstein *et al.* (1995 ▶). For the biological properties of acetophenone derivatives, see Favier *et al.* (1998 ▶); Sala *et al.* (2001 ▶); Suksamrarn *et al.* (1997 ▶). Acetophenones are useful synthons for the preparation of a wide variety of polyphenolic compounds such as chalcones and flavones, see Parmar *et al.* (1996 ▶). 
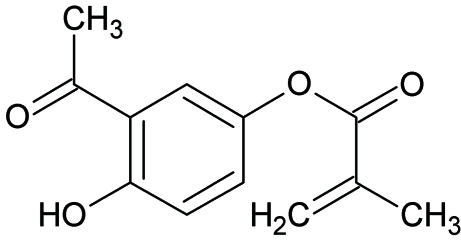


## Experimental

### 

#### Crystal data

C_12_H_12_O_4_
*M* *_r_* = 220.22
Monoclinic, 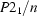

*a* = 8.8335 (3) Å
*b* = 11.9320 (3) Å
*c* = 11.3295 (3) Å
β = 111.277 (2)°
*V* = 1112.75 (6) Å^3^
*Z* = 4
Mo *K*α radiation
μ = 0.10 mm^−1^
*T* = 293 K
0.25 × 0.17 × 0.17 mm

#### Data collection

Bruker Kappa APEXII CCD diffractometer
Absorption correction: multi-scan (*SADABS*; Bruker, 2004 ▶) *T*_min_ = 0.976, *T*_max_ = 0.983
14184 measured reflections
3437 independent reflections
2080 reflections with *I* > 2σ(*I*)
*R*_int_ = 0.026

#### Refinement

*R*[*F* ^2^ > 2σ(*F* ^2^)] = 0.048
*wR*(*F* ^2^) = 0.161
*S* = 1.05
3437 reflections
155 parameters
H atoms treated by a mixture of independent and constrained refinement
Δρ_max_ = 0.21 e Å^−3^
Δρ_min_ = −0.17 e Å^−3^

### 

Data collection: *APEX2* (Bruker, 2004 ▶); cell refinement: *SAINT* (Bruker, 2004 ▶); data reduction: *SAINT* and *XPREP* (Bruker, 2004 ▶); program(s) used to solve structure: *SHELXS97* (Sheldrick, 2008 ▶); program(s) used to refine structure: *SHELXL97* (Sheldrick, 2008 ▶); molecular graphics: *ORTEP-3 for Windows* (Farrugia, 1997 ▶); software used to prepare material for publication: *SHELXL97* and *PLATON* (Spek, 2009 ▶).

## Supplementary Material

Crystal structure: contains datablocks global, I. DOI: 10.1107/S1600536809032176/wn2339sup1.cif

Structure factors: contains datablocks I. DOI: 10.1107/S1600536809032176/wn2339Isup2.hkl

Additional supplementary materials:  crystallographic information; 3D view; checkCIF report

## Figures and Tables

**Table 1:** Hydrogen-bond geometry (Å, °)

*D*—H⋯*A*	*D*—H	H⋯*A*	*D*⋯*A*	*D*—H⋯*A*
O2—H2⋯O1	0.82	1.82	2.546 (2)	146
C5—H5*A*⋯O1^i^	0.93	2.57	3.483 (2)	166
C11—H11*B*⋯O4^i^	0.96	2.57	3.336 (2)	137

Symmetry code: (i) 
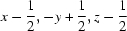
.

**Fig. 1.**
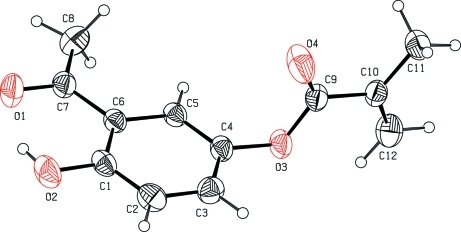
The molecular structure of the title compound, with dispacement ellipsoids drawn at the 30% probability level. Hydrogen atoms are shown as spheres of arbitrary radius.

**Fig. 2.**
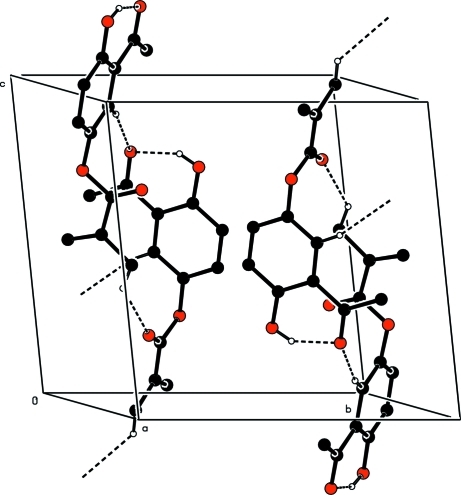
The packing of the molecules in the crystal structure. Dashed lines indicate hydrogen bonds. Hydrogen atoms not involved in hydrogen bonding have been omitted.

## supplementary crystallographic information

### Comment

Acetophenones are useful synthons for the preparation of a wide variety of polyphenolic compounds such as chalcones and flavones (Parmar *et al.*, 1996). Acetophenone derivatives have shown many interesting biological properties such as anti-inflammatory (Sala *et al.*, 2001; Favier *et al.*, 1998), cytotoxic and choleretic (Suksamrarn *et al.*, 1997) activities. Acetophenone is also used as a solvent for cellulose ethers and esters for the production of alcohol-soluble resins. 2-Hydroxy-4- methoxybenzophenone is used on an industrial scale as an ultraviolet absorber in cosmetics and plastics. 2-Hydroxyl-4,6-dimethoxyacetophenone was isolated from the leaves of the peperomia glabella family. Peperomia glabella is an epiphyte used in Venezuelan folk medicine as an anti-asthmatic.

The bond lengths C7—C8, C9—C10 and C10—C11 [1.495 (1), 1.476 (2) and 1.479 (1) Å] are comparable with standard values (Allen *et al.*, 1987). The carbonyl group bond length C7—O1 [1.235 (2) Å] is longer than C9—O4 [1.185 (2) Å]. This may be a result of O1 being involved in intramolecular and intermolecular hydrogen bonds; this would tend to lengthen the C7—O1 bond.

O2, C7, O1 and C8 are coplanar with the benzene ring. The angle between the benzene ring and the plane through O3, C9, O4, C10, C11 and C12 is 86.1 (1)° (Fig. 1).

The molecular structure of the compound is stabilized by a weak intramolecular O—H···O hydrogen bond and the crystal packing is stabilized by intermolecular C—H···O hydrogen bonds. The molecule at (*x*, *y*, *z*) is linked to the symmetry-related molecule at (-1/2 + *x*, 1/2 - *y*, -1/2 + *z*), forming a dimer with graph set descriptor *R*_2_^2^(16) (Bernstein *et al.*, 1995). Propagation of these dimer units generates an infinite molecular chain along the crystallographic *c* axis. Fig. 2 shows the crystal packing of the compound, viewed approximately down the *a* axis.

### Experimental

2,5-Dihydroxyacetophenone (26.31 mmol, 4.0 g), K_2_CO_3_ (31.55 mmol, 4.36 g) and 150 ml of dry acetone were taken up in a 250 ml round bottomed flask and the temperature was maintained at 0 °C. A solution of methacryloyl chloride (26.80 mmol, 2.8 ml) in 20 ml of dry acetone was then added dropwise to the mixture, with constant stirring for 30 min. After the addition was complete the reaction mixture was stirred for another 6 h. The salt formed during the reaction was filtered and the filtrate was washed with water and dried over anhydrous MgSO_4_. The filtrate was concentrated under reduced pressure and the crude product was purified by column chromatography (silica) using a hexane/ethyl acetate mixture (90:10). The product was collected and recrystallized from chloroform to give a crystalline white solid. Yield: 4.5 g (77%); Mp: 65–66 °C.

### Refinement

The H atoms attached to C12 were located in a difference map and refined freely. Other H atoms were positioned geometrically and were treated as riding on their parent atoms, with aromatic C—H distances of 0.93 Å, methyl C—H distances of 0.96 Å; *U*_iso_(H) = 1.5*U*_eq_(C) for methyl H and 1.2*U*_eq_(C) for aromatic H atoms. O—H = 0.82 Å and the isotropic dispacement parameter was refined. The methylene group was free to rotate, but not to tip.

### Crystal data

**Table:**

C_12_H_12_O_4_	*F*(000) = 464
*M**_r_* = 220.22	*D*_x_ = 1.315 Mg m^−^^3^
Monoclinic, *P*2_1_/*n*	Mo *K*α radiation, λ = 0.71073 Å
Hall symbol: -P 2yn	Cell parameters from 3437 reflections
*a* = 8.8335 (3) Å	θ = 2.5–30.6°
*b* = 11.9320 (3) Å	µ = 0.10 mm^−^^1^
*c* = 11.3295 (3) Å	*T* = 293 K
β = 111.277 (2)°	Block, colourless
*V* = 1112.75 (6) Å^3^	0.25 × 0.17 × 0.17 mm
*Z* = 4	

### Data collection

**Table:**

Bruker Kappa APEXII CCD diffractometer	3437 independent reflections
Radiation source: fine-focus sealed tube	2080 reflections with *I* > 2σ(*I*)
graphite	*R*_int_ = 0.026
ω and φ scans	θ_max_ = 30.6°, θ_min_ = 2.5°
Absorption correction: multi-scan (*SADABS*; Bruker, 2004)	*h* = −12→12
*T*_min_ = 0.976, *T*_max_ = 0.983	*k* = −17→12
14184 measured reflections	*l* = −15→16

### Refinement

**Table:**

Refinement on *F*^2^	Secondary atom site location: difference Fourier map
Least-squares matrix: full	Hydrogen site location: inferred from neighbouring sites
*R*[*F*^2^ > 2σ(*F*^2^)] = 0.048	H atoms treated by a mixture of independent and constrained refinement
*wR*(*F*^2^) = 0.161	*w* = 1/[σ^2^(*F*_o_^2^) + (0.0732*P*)^2^ + 0.1247*P*] where *P* = (*F*_o_^2^ + 2*F*_c_^2^)/3
*S* = 1.05	(Δ/σ)_max_ < 0.001
3437 reflections	Δρ_max_ = 0.21 e Å^−^^3^
155 parameters	Δρ_min_ = −0.17 e Å^−^^3^
0 restraints	Extinction correction: *SHELXL97* (Sheldrick, 2008), Fc^*^=kFc[1+0.001xFc^2^λ^3^/sin(2θ)]^-1/4^
Primary atom site location: structure-invariant direct methods	Extinction coefficient: 0.007 (4)

### Special details

**Table:**

Geometry. All e.s.d.'s (except the e.s.d. in the dihedral angle between two l.s. planes) are estimated using the full covariance matrix. The cell e.s.d.'s are taken into account individually in the estimation of e.s.d.'s in distances, angles and torsion angles; correlations between e.s.d.'s in cell parameters are only used when they are defined by crystal symmetry. An approximate (isotropic) treatment of cell e.s.d.'s is used for estimating e.s.d.'s involving l.s. planes.
Refinement. Refinement of *F*^2^ against ALL reflections. The weighted *R*-factor *wR* and goodness of fit *S* are based on *F*^2^, conventional *R*-factors *R* are based on *F*, with *F* set to zero for negative *F*^2^. The threshold expression of *F*^2^ > σ(*F*^2^) is used only for calculating *R*-factors(gt) *etc*. and is not relevant to the choice of reflections for refinement. *R*-factors based on *F*^2^ are statistically about twice as large as those based on *F*, and *R*- factors based on ALL data will be even larger.

### Fractional atomic coordinates and isotropic or equivalent isotropic displacement parameters (Å^2^)

**Table:**

	*x*	*y*	*z*	*U*_iso_*/*U*_eq_	
C1	0.79745 (16)	0.06020 (12)	1.12189 (13)	0.0535 (3)	
C2	0.82796 (18)	−0.01422 (13)	1.03977 (15)	0.0636 (4)	
H2A	0.9148	−0.0638	1.0702	0.076*	
C3	0.73129 (18)	−0.01579 (13)	0.91355 (14)	0.0610 (4)	
H3A	0.7534	−0.0653	0.8584	0.073*	
C4	0.60112 (16)	0.05669 (12)	0.86937 (12)	0.0508 (3)	
C5	0.56645 (15)	0.12991 (10)	0.94908 (12)	0.0468 (3)	
H5A	0.4769	0.1771	0.9177	0.056*	
C6	0.66479 (15)	0.13418 (10)	1.07749 (11)	0.0455 (3)	
C7	0.63168 (17)	0.21260 (12)	1.16503 (13)	0.0563 (4)	
C8	0.4888 (2)	0.28945 (14)	1.11997 (18)	0.0747 (5)	
H8A	0.4860	0.3344	1.1894	0.112*	
H8B	0.3908	0.2461	1.0869	0.112*	
H8C	0.4977	0.3372	1.0546	0.112*	
C9	0.51842 (17)	0.12501 (12)	0.66068 (12)	0.0539 (3)	
C10	0.39516 (16)	0.11754 (12)	0.53150 (12)	0.0524 (3)	
C11	0.4200 (2)	0.19460 (15)	0.43794 (15)	0.0741 (5)	
H11A	0.4197	0.2706	0.4657	0.111*	
H11B	0.3341	0.1846	0.3573	0.111*	
H11C	0.5225	0.1786	0.4301	0.111*	
C12	0.2721 (2)	0.04683 (17)	0.50538 (18)	0.0744 (5)	
O1	0.72227 (16)	0.21560 (11)	1.27744 (10)	0.0806 (4)	
O2	0.89890 (14)	0.05916 (12)	1.24440 (10)	0.0777 (4)	
H2	0.8702	0.1069	1.2839	0.131 (11)*	
O3	0.49615 (13)	0.05008 (9)	0.74249 (9)	0.0621 (3)	
O4	0.62770 (17)	0.18962 (14)	0.69180 (11)	0.1046 (5)	
H12A	0.195 (3)	0.0427 (17)	0.420 (2)	0.101 (6)*	
H12B	0.257 (3)	−0.0033 (19)	0.569 (2)	0.107 (7)*	

### Atomic displacement parameters (Å^2^)

**Table:**

	*U*^11^	*U*^22^	*U*^33^	*U*^12^	*U*^13^	*U*^23^
C1	0.0459 (7)	0.0615 (8)	0.0460 (7)	−0.0046 (6)	0.0081 (6)	0.0116 (6)
C2	0.0517 (8)	0.0687 (9)	0.0676 (9)	0.0109 (7)	0.0184 (7)	0.0115 (7)
C3	0.0638 (9)	0.0606 (9)	0.0632 (9)	0.0001 (7)	0.0287 (7)	−0.0031 (7)
C4	0.0518 (7)	0.0558 (7)	0.0411 (6)	−0.0130 (6)	0.0123 (6)	−0.0005 (5)
C5	0.0423 (6)	0.0496 (7)	0.0429 (6)	−0.0039 (5)	0.0090 (5)	0.0045 (5)
C6	0.0446 (6)	0.0482 (7)	0.0405 (6)	−0.0071 (5)	0.0114 (5)	0.0037 (5)
C7	0.0626 (8)	0.0581 (8)	0.0460 (7)	−0.0127 (6)	0.0171 (6)	−0.0022 (6)
C8	0.0814 (11)	0.0673 (10)	0.0778 (11)	0.0019 (8)	0.0316 (9)	−0.0141 (8)
C9	0.0565 (8)	0.0608 (8)	0.0429 (7)	−0.0084 (6)	0.0165 (6)	−0.0062 (6)
C10	0.0540 (7)	0.0572 (8)	0.0427 (7)	0.0101 (6)	0.0137 (6)	−0.0055 (5)
C11	0.0910 (12)	0.0762 (10)	0.0522 (9)	0.0150 (9)	0.0222 (8)	0.0069 (7)
C12	0.0591 (9)	0.0910 (13)	0.0576 (9)	−0.0046 (9)	0.0027 (8)	−0.0060 (9)
O1	0.0927 (9)	0.0936 (9)	0.0455 (6)	−0.0097 (7)	0.0130 (6)	−0.0124 (5)
O2	0.0672 (7)	0.0942 (9)	0.0505 (6)	0.0060 (6)	−0.0040 (5)	0.0160 (6)
O3	0.0678 (6)	0.0697 (7)	0.0405 (5)	−0.0222 (5)	0.0098 (4)	−0.0036 (4)
O4	0.1082 (10)	0.1330 (12)	0.0555 (7)	−0.0691 (9)	0.0092 (7)	0.0069 (7)

### Geometric parameters (Å, °)

**Table:**

C1—O2	1.352 (2)	C8—H8A	0.9600
C1—C2	1.382 (2)	C8—H8B	0.9600
C1—C6	1.4062 (19)	C8—H8C	0.9600
C2—C3	1.374 (2)	C9—O4	1.185 (2)
C2—H2A	0.9300	C9—O3	1.353 (2)
C3—C4	1.379 (2)	C9—C10	1.476 (2)
C3—H3A	0.9300	C10—C12	1.322 (2)
C4—C5	1.3688 (19)	C10—C11	1.479 (2)
C4—O3	1.402 (2)	C11—H11A	0.9600
C5—C6	1.3987 (17)	C11—H11B	0.9600
C5—H5A	0.9300	C11—H11C	0.9600
C6—C7	1.4681 (19)	C12—H12A	0.96 (2)
C7—O1	1.235 (2)	C12—H12B	0.98 (2)
C7—C8	1.492 (2)	O2—H2	0.8200
			
O2—C1—C2	117.83 (13)	C7—C8—H8B	109.5
O2—C1—C6	121.95 (14)	H8A—C8—H8B	109.5
C2—C1—C6	120.22 (12)	C7—C8—H8C	109.5
C3—C2—C1	120.68 (13)	H8A—C8—H8C	109.5
C3—C2—H2A	119.7	H8B—C8—H8C	109.5
C1—C2—H2A	119.7	O4—C9—O3	122.11 (13)
C2—C3—C4	119.37 (14)	O4—C9—C10	124.24 (13)
C2—C3—H3A	120.3	O3—C9—C10	113.65 (12)
C4—C3—H3A	120.3	C12—C10—C9	120.75 (14)
C5—C4—C3	121.16 (12)	C12—C10—C11	124.09 (15)
C5—C4—O3	119.30 (12)	C9—C10—C11	115.16 (13)
C3—C4—O3	119.41 (12)	C10—C11—H11A	109.5
C4—C5—C6	120.41 (12)	C10—C11—H11B	109.5
C4—C5—H5A	119.8	H11A—C11—H11B	109.5
C6—C5—H5A	119.8	C10—C11—H11C	109.5
C5—C6—C1	118.14 (12)	H11A—C11—H11C	109.5
C5—C6—C7	121.67 (12)	H11B—C11—H11C	109.5
C1—C6—C7	120.19 (12)	C10—C12—H12A	118.7 (13)
O1—C7—C6	120.13 (14)	C10—C12—H12B	123.1 (13)
O1—C7—C8	119.10 (14)	H12A—C12—H12B	118.2 (19)
C6—C7—C8	120.78 (13)	C1—O2—H2	109.5
C7—C8—H8A	109.5	C9—O3—C4	117.32 (10)
			
O2—C1—C2—C3	−178.7 (1)	C5—C6—C7—O1	−178.84 (13)
C6—C1—C2—C3	1.1 (2)	C1—C6—C7—O1	1.6 (2)
C1—C2—C3—C4	−1.0 (2)	C5—C6—C7—C8	1.2 (2)
C2—C3—C4—C5	−0.1 (2)	C1—C6—C7—C8	−178.37 (13)
C2—C3—C4—O3	−175.79 (12)	O4—C9—C10—C12	−176.71 (18)
C3—C4—C5—C6	1.18 (19)	O3—C9—C10—C12	3.5 (2)
O3—C4—C5—C6	176.83 (11)	O4—C9—C10—C11	2.6 (2)
C4—C5—C6—C1	−1.04 (18)	O3—C9—C10—C11	−177.24 (12)
C4—C5—C6—C7	179.36 (11)	O4—C9—O3—C4	4.5 (2)
O2—C1—C6—C5	179.7 (1)	C10—C9—O3—C4	−175.63 (11)
C2—C1—C6—C5	−0.10 (19)	C5—C4—O3—C9	84.7 (2)
O2—C1—C6—C7	−0.69 (19)	C3—C4—O3—C9	−99.6 (2)
C2—C1—C6—C7	179.50 (12)		

### Hydrogen-bond geometry (Å, °)

**Table:**

*D*—H···*A*	*D*—H	H···*A*	*D*···*A*	*D*—H···*A*
O2—H2···O1	0.82	1.82	2.546 (2)	146
C5—H5A···O1^i^	0.93	2.57	3.483 (2)	166
C11—H11B···O4^i^	0.96	2.57	3.336 (2)	137

Symmetry codes: (i) *x*−1/2, −*y*+1/2, *z*−1/2.
